# Estimating complete cancer prevalence in Europe: validity of alternative vs standard completeness indexes

**DOI:** 10.3389/fonc.2023.1114701

**Published:** 2023-04-24

**Authors:** Elena Demuru, Silvia Rossi, Leonardo Ventura, Luigino Dal Maso, Stefano Guzzinati, Alexander Katalinic, Sebastien Lamy, Valerie Jooste, Corrado Di Benedetto, Roberta De Angelis, the EUROCARE-6 Working Group, M. Hackl

**Affiliations:** ^1^ Department of Oncology and Molecular Medicine, Istituto Superiore di Sanità, Rome, Italy; ^2^ Clinical and Descriptive Epidemiology Unit, Istituto per lo Studio, la Prevenzione e la Rete Oncologica (ISPRO), Firenze, Italy; ^3^ Cancer Epidemiology Unit, Centro di Riferimento Oncologico (CRO), Istituto di Ricerca e Cura a Carattere Scientifico (IRCCS), Aviano, Italy; ^4^ Veneto Cancer Registry, Azienda Zero, Padova, Italy; ^5^ Cancer Registry of Schleswig-Holstein, Institute for Social Medicine and Epidemiology, University of Lübeck, Lübeck, Germany; ^6^ Tarn Cancer Registry, Claudius Regaud Institute - Center for Epidemiology and Research in Population Health (CERPOP U1295), University of Toulouse - Inserm, Toulouse, France; ^7^ Digestive Cancer Registry of Burgundy, Dijon University Hospital, INSERM UMR1231, Dijon, France; ^8^ IT Service, Istituto Superiore di Sanità, Rome, Italy

**Keywords:** cancer prevalence, cancer registries, cancer survivors, cancer survivorship, EUROCARE, Europe, SEER program

## Abstract

**Introduction:**

Comparable indicators on complete cancer prevalence are increasingly needed in Europe to support survivorship care planning. Direct measures can be biased by limited registration time and estimates are needed to recover long term survivors. The completeness index method, based on incidence and survival modelling, is the standard most validated approach.

**Methods:**

Within this framework, we consider two alternative approaches that do not require any direct modelling activity: i) empirical indices derived from long established European registries; ii) pre-calculated indices derived from US-SEER cancer registries. Relying on the EUROCARE-6 study dataset we compare standard vs alternative complete prevalence estimates using data from 62 registries in 27 countries by sex, cancer type and registration time.

**Results:**

For tumours mostly diagnosed in the elderly the empirical estimates differ little from standard estimates (on average less than 5% after 10-15 years of registration), especially for low prognosis cancers. For early-onset cancers (bone, brain, cervix uteri, testis, Hodgkin disease, soft tissues) the empirical method may produce substantial underestimations of complete prevalence (up to 20%) even when based on 35-year observations. SEER estimates are comparable to the standard ones for most cancers, including many early-onset tumours, even when derived from short time series (10-15 years). Longer observations are however needed when cancer-specific incidence and prognosis differ remarkably between US and European populations (endometrium, thyroid or stomach).

**Discussion:**

These results may facilitate the dissemination of complete prevalence estimates across Europe and help bridge the current information gaps.

## 1 Introduction

Cancer prevalence statistics enumerate the number, or the proportion, of people in a population living after a cancer diagnosis at a specific date. Unlike other surveillance metrics based on cancer registries’ observations, such as incidence or survival, direct measures of prevalence are intrinsically incomplete, as they cannot include the cancer survivors diagnosed before the start of registration. Complete prevalence must be necessarily estimated to recover long term survivors, especially when the period of registration is limited.

The completeness index method is one of the most accurate and used methods to estimate complete prevalence starting from limited-duration prevalence measured by cancer registries (1). Based on incidence and relative survival modelling and on their relationship with prevalence, this method provides a correction factor, the so-called completeness index, or R-index, to complete cancer-specific registries observations.

The completeness index method has been systematically validated and applied since many years in the USA (2), where complete prevalence statistics are published annually as an integral part of the SEER Cancer Statistics (3). A software to implement the method is distributed by the National Cancer Institute, along with completeness indexes derived from the SEER registries datasets (4).

Conversely, in Europe complete prevalence estimates are not systematically available in all countries with active population-based cancer registries. European cancer prevalence estimates by country are made available by GLOBOCAN (5), however they are limited to 5-years since diagnosis (6). Occasionally, on a project basis, the completeness index method has been applied to European CRs data to derive complete prevalence of rare cancers (7–9) or frequent cancers by European country and area (10, 11). Complete prevalence is periodically estimated through the completeness index approach only in Italy (12, 13), where the method was first proposed. Experiences in other countries refer to limited-duration prevalence (14) or to different methods (15–19). Only some European registries operating since the 50s, such as those in Nordic countries or Slovenia, are able to measure a virtually complete prevalence without any estimation (20, 21).

Integrating traditional surveillance metrics with accurate complete prevalence estimates is of increasing importance, given the remarkable growth of cancer survivors in all ageing societies. They represent a heterogeneous population, in terms of healthcare needs and quality of life, that should be better quantified and qualified (22–27). Given this background, closing the existing gaps in Europe is one of the priorities in cancer surveillance.

Promoting the use and dissemination of complete cancer prevalence indicators by country in Europe was one of the goals of the European Joint Action on Cancer iPAAC (Innovative Partnership for Action Against Cancer) (28). Exploring the feasibility of viable solutions to facilitate the use of completeness indexes was part of the project’s activities.

With this purpose, in the present study we compared the standard method of deriving prevalence completeness index in Europe (by modelling incidence and survival data from European populations) with alternative approaches that do not require any statistical modelling, namely: i) empirical indexes derived from the longest prevalence data available from European registries; ii) publicly available model-based indexes estimated from SEER-US data (4). The study aims to assess under which conditions of application (registration time length and cancer type) these “non-standard” approaches may adequately surrogate the reference method, which remains the “gold standard”.

Nowadays, indeed, cancer prevalence observations are available for time series and populations to a much greater extent than when R-indexes were first proposed (1). Assessing application conditions of empirical R-indexes may facilitate the use and dissemination of complete prevalence estimates across Europe and contribute to bridge the present information gaps. For the same reasons it is worth exploring the application limits to European data of SEER-US indexes that are publicly available and ready to be used.

## 2 Materials and methods

The study relies on the dataset of the EUROCARE-6 project, a wide collaborative study on cancer survival and prevalence in Europe (29) based on cancer registries data. The dataset includes pseudonymised individual data on cancer patients’ incidence and life status, as well as life tables and resident population in each registry.

For the purpose of the study we selected 62 general cancer registries from 27 European countries (21 with national population coverage) providing prevalence data up to 1/1/2013, the most recent common prevalence index date available in the dataset. At this date the maximum duration of registration ranged from 5 to 35 years, with median at 20 years.

The following four different types of analyses were conducted each using a specific dataset depending on the scope. Cancer registries included 5% to 50% coverage of the 27 countries’ population (**Table 1**).

a) *Empirical completeness indexes*. Pooled prevalence data from 8 registries with an observation period of 35 years (maximum available duration of registration) were used to estimate European empirical completeness indexes.
b) *Model-based completeness indexes*. Pooled incidence and relative survival data from 11 registries with at least 30 years of observation were used to derive standard European model-based completeness indexes.
c) *Validation of completeness indexes*. Registry-specific prevalence from the registries with at least 20 years of observation were the reference to validate European model-based completeness indexes (gold-standard method) estimated in step b. Registries in dataset b) were excluded from the validation dataset.
d) *Complete prevalence estimation*. Registry-specific observed prevalence from all eligible 62 registries, up to their maximum registration duration (from 5 to 35 years), were used to estimate complete prevalence in each registry according to standard and alternative methods.

**Table 1:** Description of the registries included in each analysis-specific dataset.

Dataset	Type of analysis	Registration length (years)	Number of registries	Registries	Population (% study coverage)
a)	Empirical index	35	8	**Denmark, Estonia, Finland, Iceland, Norway, Scotland,** Geneva *(Switzerland)*, Parma *(Italy)*	23,592,911 (5%)
b)	Model-based index	>=30	11	*Registries in dataset a) plus:* ***Austria, Slovenia*,** Tarragona (Spain)*	34,806,065 (8%)
c)	Validation of completeness indexes	>=20	20	**Bulgaria, Lithuania, Malta, The Netherlands, Northern Ireland, Wales,** Balearic Islands, Basque Country, Granada *(Spain)*, Graubünden and Glarus, Eastern Switzerland *(Switzerland)*, Bas Rhin, Doubs, Haut-Rhin, Isere, Somme, Tarn *(France)*, Modena, Ragusa, Romagna *(Italy)*	44,230,482 (10%)
d)	Complete prevalence estimation	>=5	62	*Registries in dataset c) and b) plus:* **Belgium, Cyprus, Czech Republic, England, Ireland, Latvia, Poland,** Herault, Lille, Poitou Charentes *(France)*, Bremen, Federal States (BR,MW-PSA,THU), Hamburg *(Germany)*, Bergamo, Puglia Barletta Andria-Trani, Catania-Messina-Enna, Latina, Monza-Brianza, Napoli, Nuoro, Palermo, Piacenza, Reggio Emilia, Siracusa, Sondrio, Taranto, Umbria *(Italy)*, Southern Portugal *(Portugal)*, Castellon, Girona *(Spain)*, Friburg, Ticino *(Switzerland)*	231,214,391 (51%)

*The registry of Tarragona is included in dataset b) and not in d) because limited-duration prevalence is available at 1/1/2012.

Population covered by the registries in each dataset and percent coverage of the 27 European countries that participated in EUROCARE-6 included in the study are shown. National registries are in bold.

To compare complete prevalence values estimated from the different completeness indexes we performed distinct analyses for a selection of 30 common index cancers. Cancer entities were defined according to the Third Revision of the International Classification of Diseases for Oncology (ICDO-3). Only malignant primary cancers were included, except for brain and urinary bladder (**Supplementary Materials**, **Table A1**). Non-malignant tumours proportion by registry ranges from 0 to 28% for brain cancer and from 0 to 54% for urinary bladder, thus reflecting varying registration criteria across Europe. The first primary tumour for each cancer entity was considered, meaning that each person was counted only once and that people with multiple primary cancers affecting different sites contribute to prevalence counts of different entities. Consequently, cancer-specific counts do not sum up to counts of all cancers combined.

### 2.1 Observed limited-duration prevalence

Limited-duration prevalence observed in each registry population was computed at the index date with the counting method, available in the SEER*Stat software (30) by enumerating the number of patients known to be alive at the index date. Life-table survival probabilities stratified by registry, sex, grouped age at diagnosis (0-59, 60-74, 75+), cancer site and 5-year period of diagnosis, were attributed to patients lost to follow-up to count those estimated alive at the prevalence index date. Age at the prevalence date was detailed in 5-year groups and 85+. The proportion of lost to follow-up is generally very low, below 2% in most countries.

### 2.2 Completeness index estimation (R-index)

R-index at duration *d* (R_d_) is defined as the ratio of prevalence at duration *d* to estimated complete prevalence. It expresses an estimation of percent completeness of a given limited-duration prevalence. Complete prevalence is therefore estimated dividing the number of observed prevalent cases at a given duration *d* (N_d_) by the corresponding R-index at the same duration (1).

For each cancer we derived R-index by sex, age at prevalence date ( i ) in 5-year age groups and annual registration duration (*d*). Model-based and empirical approaches were both considered.

i) European empirical R-index (EU emp)
Empirical R-indexes were obtained from the pool of registries in dataset a) (**Table 1**) as the ratio of the observed prevalent cases at duration d to the observed prevalent cases at the maximum duration (35 years), namely $R_{i,d}=N_{i,d}/N_{i,35}$ . Age at prevalence date was grouped in 5-year classes except for extreme ages (0-29 and 80+) for which wider groupings were used to avoid random fluctuations due to the scarce number of cases. Using these empirical indexes is to assume that observed 35-year limited duration prevalence equals (i.e. is sufficiently close to) complete prevalence.
ii) Standard European model-based R-index (EU mod)
For the pool of registries in dataset b) (**Table 1**) we computed incidence rates and relative survival (RS) with the SEER*Stat software (30). RS, the ratio of observed survival in a group of cancer patients to the expected survival in a comparable group from the general population, was determined using the Ederer 2 cohort method. Incidence and survival data were stratified by cancer type, sex, 5-year period of diagnosis (1980-1984, 1985-1989, 1990-1994, 1995-1999, 2000-2004, 2005-2009, 2010-2014) and age at diagnosis (5-year and 85+ for incidence; cancer-specific strata for relative survival are given in **Table A1** **Supplementary Materials**). We modelled pooled incidence and relative survival data following the standard methodology (2). We fitted a mixture “cure-model” of Weibull type to RS data. These models assume that only a fraction of patients will die of the disease, with time to death following a Weibull distribution, while the others are considered as cured. The non-linear regression procedure (NLIN) available in the SAS Software (SAS System for Windows, version 9.4; SAS Institute, Cary, NC) was used to estimate model parameters.
We fitted two alternative logistic age-cohort models to incidence rates stratified by age and period of diagnosis. Non-parametric cohort-effect was modelled through 10-year groups and parametric dependency on age at diagnosis was assumed by using respectively an exponential or a six-degree polynomial. Both models were estimated with the SAS LOGISTIC procedure.
Parameters of survival and incidence models were then imported in the software implementing the completeness index standard method (COMPREV) (4) to produce European model-based R-indexes.
iii) SEER model-based R-index (SEER mod)
Model-based R-indexes, estimated by the US National Cancer Institute (NCI) from the SEER-Program cancer registries data, were extracted from the COMPREV software (4).

### 2.3 Validation of the completeness indexes

The completeness index method allows to estimate any limited-duration prevalence beyond the longest observed period. Prevalence at any duration d_2_ can be estimated dividing observed prevalence at maximum available duration d_1_ by the ratio of the two corresponding R-indexes: R_d1_/R_d2_.

We used this property to validate R-indexes estimated by modelling European data, i.e. by using the gold standard method. For each eligible registry observed, 20-year prevalence was compared with estimated 20-year prevalence. To simulate a registration activity shorter than 20 years, observed prevalence was artificially truncated at durations d=5,10,15 years. The goodness of fit was measured separately for each cancer type as the weighted average percent relative difference in absolute value between estimated (N’) and observed (N) 20-year number of prevalent cases ( APRD ):


$$\;APRD=\sum_{r}\left(\frac{\left|N_{20,r}^{'}-N_{20,r}\right|}{N_{20,r}}\right)w_{r}\times 100$$

Registry-specific proportions of cancer cases (*w_r_*) were used as weights. The absolute value of the relative difference avoids compensations between under- and over-estimations and provides a maximum average discrepancy compared to observations. The registries used for this validation (dataset c in **Table 1**) did not coincide with those used for estimating European model-based R-indexes (dataset b in **Table 1**).

### 2.4 Comparison of complete prevalence estimates

Cancer-, sex-, age- and duration-specific prevalence completeness indexes were applied to observed prevalence at maximum available duration in each of the 62 registries in dataset d) to obtain estimates of complete prevalence at 1/1/2013. Standard model-based complete prevalence estimates were compared to those obtained with alternative R-indexes (EU emp or SEER mod).

Weighted average percent relative difference between alternative and standard estimates of complete prevalence (PRD) was analysed by cancer site, sex and grouped registration duration (10-14 years, 15-19 years, 20-24 years, 25-35 years). The resident population covered by each registry was used as weight in the average.

## 3 Results

### 3.1 Incidence and relative survival models

In general, mixture cure models fitted data well and observed relative survival generally lied within the confidence limits estimated for predicted survival (examples are reported in the **Supplementary Materials**, **Figure A1**). Moreover, in most cases the survival curves reached a plateau within 20 years of follow-up, meaning that the cure assumption is satisfied in this time interval.

Diagnostic plots and values of the Akaike Information Criterion (AIC) showed that polynomial models fitted incidence data much better than exponential models for all the considered cancer types (**Supplementary Materials**, **Figure A2**). This is particularly evident for cancers at early onset or with bimodal age at diagnosis. Age polynomials provide indeed higher flexibility in modelling age trends compared to the exponential model.

### 3.2 Trends of the completeness indexes

**Figure 1:**
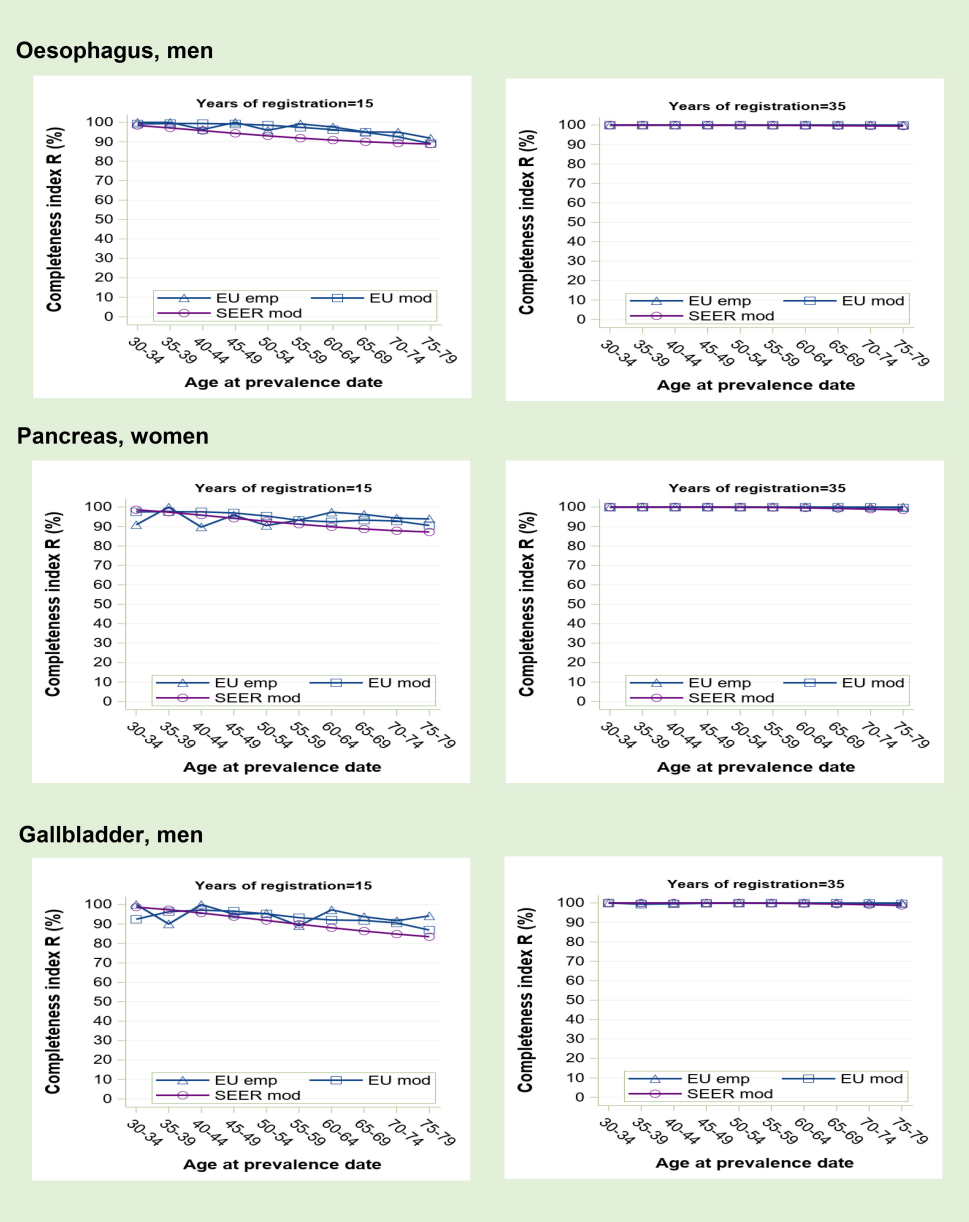
Prevalence completeness index (R index) at 1st January 2013 estimated for some tumours at low prognosis (oesophagus, pancreas, gallbladder) according to alternative methods: SEER model-based, EU model-based, EU empirical by age at prevalence date and registration time length (15 and 35 years).

**Figure 2:**
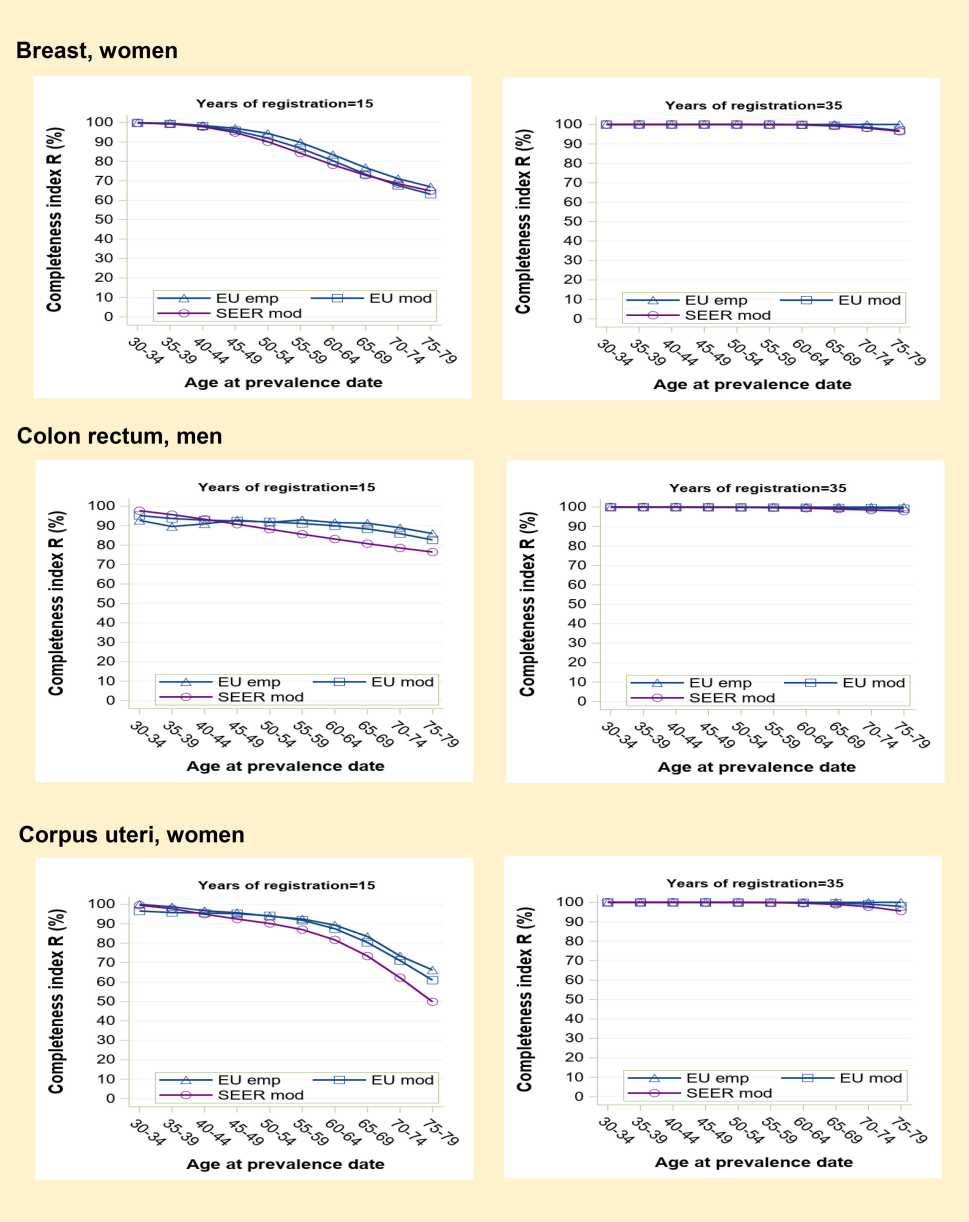
Prevalence completeness index (R-index) at 1st January 2013 estimated for some frequent medium-high prognosis tumours (breast, colon-rectum, corpus uteri) according to alternative methods: SEER model-based, EU model-based, EU empirical by age at prevalence date and registration time length (15 and 35 years).

**Figure 3:**
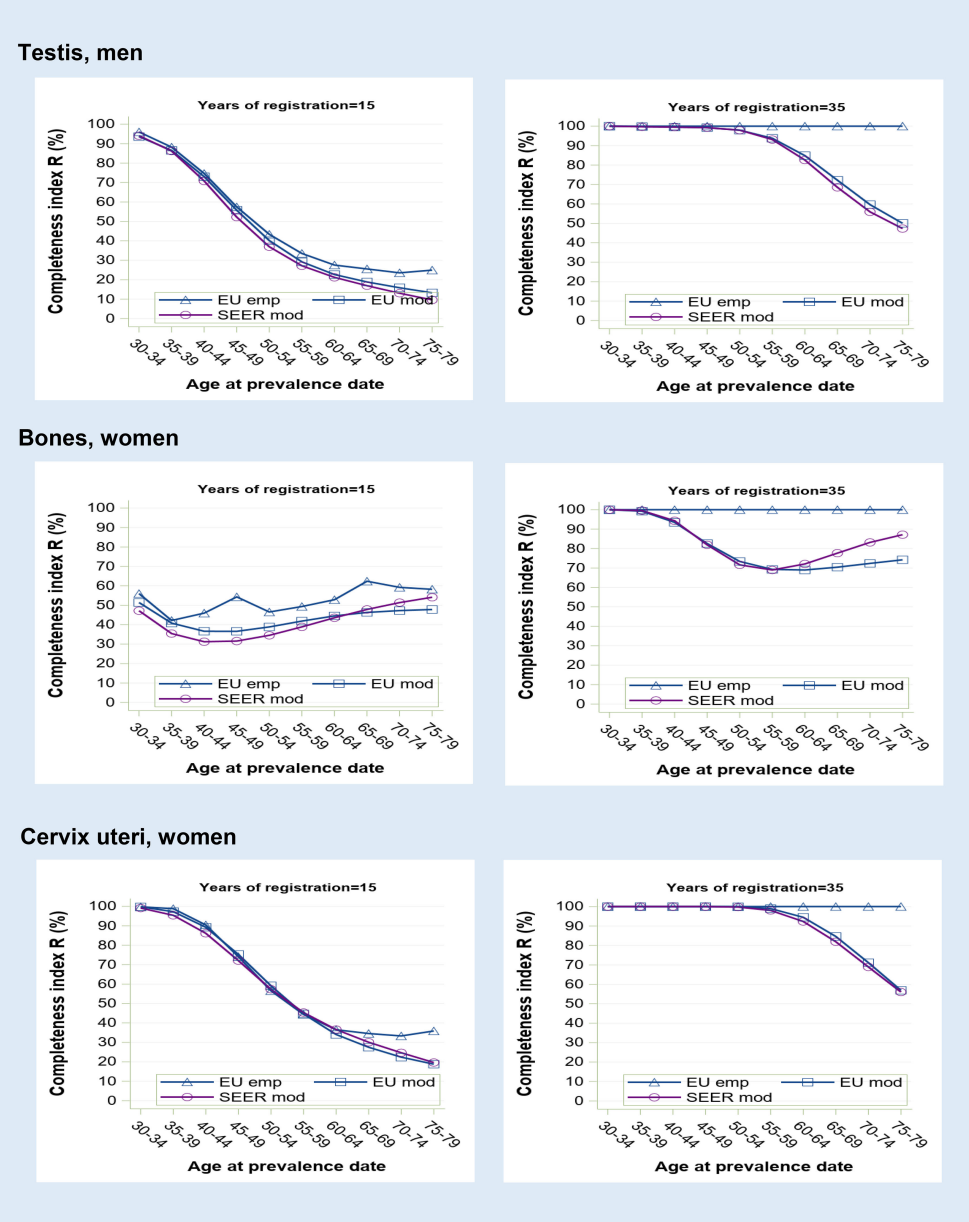
Prevalence completeness index (R-index) at 1st January 2013 estimated for some tumours diagnosed at young age (testis, bones, cervix uteri) according to alternative methods (SEER model-based, EU model-based, EU empirical by age at prevalence date and registration time length (15 and 35 years).

Some examples of cancer-specific completeness indexes trends by age at prevalence date and duration of registration are shown in **Figures 1**–**3**. The comparison of the three different methods (SEER mod, EU mod and EU Emp) is restricted to the age range 30-79 years for which R-index can be estimated for all methods by 5-year age classes. Wider groups (0-29 and 80+) are in fact needed to compute empirical indexes for extreme age ranges with few cases.

Completeness index increases with the length of registration period and is higher for cancers at low prognosis (**Figure 1**) than for those at high to medium prognosis (**Figure 2**). A reduced survival implies indeed a more complete observed prevalence. Generally, R-index is close to 100% at young age and decreases with advancing age at prevalence date. For early onset tumours (**Figure 3**), however, young survivors can be partly not observable depending on the length of registration activity. Prevalence completeness is highest for low prognosis cancers diagnosed mainly in the elderly (**Figure 1**). At 15 years of registration, R-index is above 80-90% with minimum values for the eldest survivors. The empirical index trend is less smooth compared to model-based R-indexes because, being based on observations, it is more subject to random fluctuations, as also proven by confidence intervals (not shown in the graphs). At 35-years of registration all methods provide R-index values around 100%, meaning that such duration is sufficiently long to detect practically all survivors.

Prevalence completeness is intermediate for higher prognosis cancers diagnosed in middle to old age (**Figure 2**). In the examples shown (breast, colorectal and corpus uteri cancers), at 15 years of registration, R-index varies from 95-100% to 50-70% as a function of age at prevalence date. SEER R-index values are slightly lower compared to those based on European data, reflecting a more favourable prognosis for US patients. At 35 years, model-based R-indexes tend to converge to 100% (95-98% for the eldest age group).

Cancers at early onset show the lowest R-index values and the most marked variations (**Figure 3**). At 15 years, observed prevalence is far from being complete for most age groups, particularly for bone cancers that are almost equally diagnosed at all ages. A registration period of 35 years appears insufficient to observe all long-term survivors, as shown by the residual gap (up to 50%) between empirical and model-based R-index estimates. By contrast, SEER and standard R-index, which are both model-based, show a quite similar age profile.

### 3.3 Validation of the completeness indexes

**Tables 2A, B** report observed 20-year prevalence proportion per 100,000 for the pool of registries in the validation dataset, for male and female populations, respectively. The weighted average percent relative differences, in absolute value, between registry-specific 20-year observed and standard estimated prevalence (APRD) is also reported and is obtained by artificially truncating observed prevalence at 5,10 and 15 years.

**Table 2a:** Validation of European model-based R-index, *men*.

Cancer site	Observed 20-y Prevalence	APDR, 20-y prevalence estimated by truncating registries observations at		
		15 years	10 years	5 years
	*Proportion x 100,00*	*%*	*%*	*%*
**All sites**	**2,999**	*2.0*	*5.0*	*6.4*
**Prostate**	**1,082**	*1.1*	*3.3*	*12.5*
**Colon Rectum**	**488**	*1.2*	*2.8*	*6.0*
**Bladder**	**354**	*1.1*	*2.9*	*5.5*
**Skin melanoma**	**161**	*2.1*	*5.9*	*13.8*
**Lung**	**157**	*1.3*	*3.1*	*3.3*
**Kidney**	**138**	*1.4*	*4.5*	*8.4*
**Non-Hodgkin lymphoma**	**122**	*0.9*	*3.1*	*5.7*
**Testis**	**103**	*2.1*	*5.0*	*10.9*
**Larynx**	**79**	*1.6*	*2.9*	*6.2*
**Head and neck**	**76**	*1.1*	*2.9*	*5.0*
**Stomach**	**61**	*2.0*	*4.4*	*9.7*
**CLL/SLL**	**49**	*1.6*	*4.2*	*7.5*
**Hodgkin lymphoma**	**38**	*3.1*	*5.4*	*9.3*
**Thyroid**	**33**	*4.2*	*8.3*	*14.3*
**Brain**	**32**	*4.6*	*9.5*	*13.8*
**Multiple myeloma**	**31**	*1.0*	*3.5*	*6.1*
**Soft tissues**	**30**	*2.2*	*3.1*	*4.3*
**Oesophagus**	**27**	*1.5*	*3.4*	*5.7*
**Liver**	**19**	*0.9*	*1.7*	*4.2*
**Pancreas**	**16**	*1.3*	*5.1*	*6.8*
**Penis**	**15**	*1.8*	*2.6*	*5.4*
**AML**	**12**	*2.3*	*3.0*	*4.8*
**CML**	**10**	*2.4*	*4.1*	*5.8*
**Gallbladder**	**9**	*1.3*	*4.7*	*9.6*
**Bones**	**9**	*3.0*	*6.3*	*10.4*

CLL/SLL, Chronic lymphocytic leukaemia/small lymphocytic lymphoma; AML, Acute myeloid leukaemia; CML, Chronic myeloid leukaemia.

The validation is limited to cancer registries with at least 20 years of observations at 1/1/2013 that were not used to estimate R-index. Pooled observed 20-y prevalence proportion and weighted average percent relative differences in absolute value (APRD, %) between observed and estimated 20-years prevalence by cancer site are shown. Estimates are derived by applying completeness indexes to observed prevalence truncated at 5, 10 and 15 years and the average is weighted using the registry-specific number of prevalent cases.

**Table 2b:** Validation of European model-based R-index, *women*.

Cancer site	Observed 20-y Prevalence	APDR, 20-y prevalence estimated by truncating registries observations at		
		15 years	10 years	5 years
	*Proportion x 100,00*	*%*	*%*	*%*
**All sites**	**3,471**	*1.8*	*3.9*	*5.9*
**Breast**	**1,537**	*0.9*	*1.9*	*3.5*
**Colon Rectum**	**414**	*1.4*	*3.0*	*4.8*
**Corpus Uteri**	**274**	*2.3*	*4.7*	*7.0*
**Skin melanoma**	**218**	*2.3*	*6.1*	*11.8*
**Cervix uteri**	**144**	*6.3*	*14.3*	*21.0*
**Thyroid**	**119**	*5.4*	*12.0*	*16.3*
**Ovary**	**110**	*0.7*	*1.1*	*5.7*
**Non-Hodgkin lymphoma**	**105**	*1.4*	*2.8*	*4.6*
**Bladder**	**94**	*2.0*	*4.8*	*7.7*
**Lung**	**88**	*1.3*	*3.5*	*4.8*
**Kidney**	**84**	*1.5*	*3.7*	*6.9*
**Stomach**	**41**	*2.1*	*5.5*	*9.3*
**Head and Neck**	**37**	*1.2*	*2.7*	*4.9*
**CLL/SLL**	**36**	*1.6*	*4.1*	*8.2*
**Hodgkin lymphoma**	**31**	*1.9*	*5.1*	*8.7*
**Brain**	**26**	*4.9*	*10.8*	*18.1*
**Multiple myeloma**	**26**	*1.1*	*4.1*	*7.3*
**Soft tissues**	**22**	*1.6*	*3.8*	*7.5*
**Pancreas**	**15**	*1.1*	*4.1*	*6.6*
**AML**	**12**	*2.3*	*4.0*	*8.1*
**Larynx**	**11**	*1.6*	*3.6*	*4.8*
**Oesophagus**	**10**	*2.1*	*4.0*	*5.6*
**Gallbladder**	**10**	*2.5*	*6.4*	*9.7*
**Bones**	**8**	*2.0*	*3.5*	*6.2*
**CML**	**8**	*3.1*	*5.5*	*10.7*
**Liver**	**7**	*1.5*	*3.8*	*5.2*

CLL/SLL: Chronic lymphocytic leukaemia/small lymphocytic lymphoma; AML, Acute myeloid leukaemia; CML, Chronic myeloid leukaemia.

The validation is limited to cancer registries with at least 20 years of observations at 1/1/2013 that were not used to estimate R-index. Pooled observed 20-y prevalence proportion and weighted average percent relative differences in absolute value (APRD, %) between observed and estimated 20-years prevalence by cancer site are shown. Estimates are derived by applying completeness indexes to observed prevalence truncated at 5, 10 and 15 years and the average is weighted using the registry-specific number of prevalent cases.

Average discrepancies between estimates and observations decrease as registration length increases. Particularly with registration times of 15 years the fit to observations is always good (APRD are well below 5%, maximum 6.3% for cervical cancer). At 10 years the validation is equally satisfying for all cancers examined (APRD values do not exceed 5%) except for young-onset cancers (cervix uteri, thyroid, brain and, to lesser extent, skin melanoma, bones, testis and Hodgkin lymphoma), suggesting that 15-year observed prevalence provides a more robust basis for this class of tumours.

Conversely prevalence observations limited to 5-years lead to less precise estimates in most of the cases (APRD exceed 5%) especially, but not only, for young-onset cancers (21% for cervical cancer, 12.5% for prostatic cancer).

### 3.4 Comparative assessment of complete prevalence estimates

**Figure 4:**
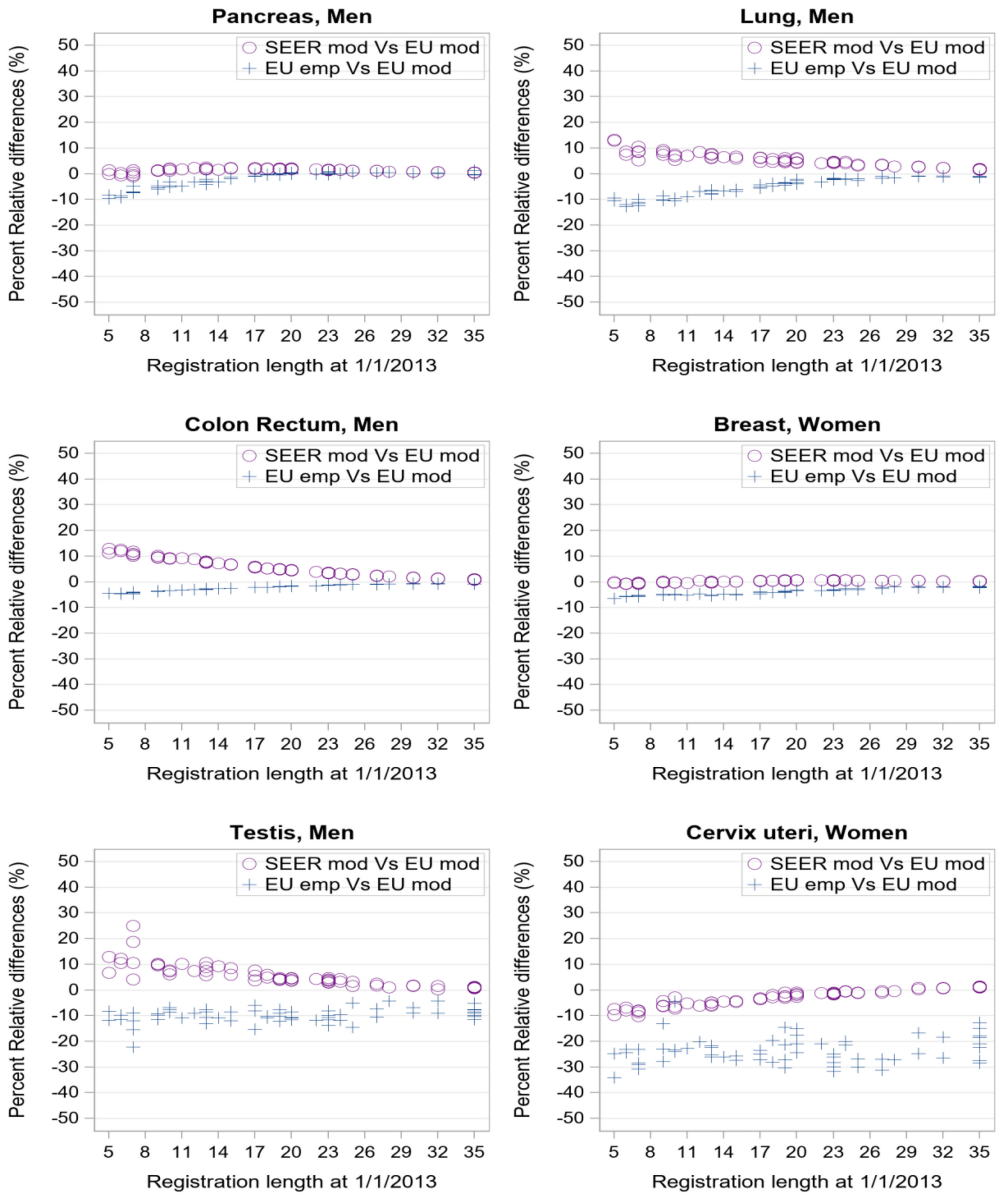
Percent relative difference (%) by registration length at 1/1/2013 of complete prevalence estimates obtained with SEER model-based or EU empirical R-index against EU model-based estimate as reference value. Each point corresponds to one of the 62 registries in dataset d of **Table 1**.

Empirical (EU Emp) and SEER (SEER mod) complete prevalence estimates were compared to the standard model-based estimates (EU mod) for all 62 eligible cancer registries (dataset d). PRD between alternative and standard complete prevalence estimates of some index tumours is plotted in **Figure 4** by registration time length (from 5 to 35 years).

Consistently with **Figures 1**, **2**, when considering cancers at late age at onset with low (pancreas, lung) or good prognosis (colon-rectum and breast), the empirical estimates (**Figure 4**, blue crosses) approach model-based estimates as registration length increases. PRD values between -5% and 0 are indeed reached already after 10 years of registration. Conversely, for testicular and cervical cancers empirical indexes provide complete prevalence estimates that are systematically lower than model-based estimates (PRD at about -10% or -20% respectively) regardless of the registration time length, consistently with R-index patterns for early-onset tumours (**Figure 3**).

Differences between SEER and standard European complete prevalence estimates (**Figure 4**, purple circles) are almost null at all durations for pancreatic and breast cancers, and after 20 years of observation, for colorectal and lung cancers. Being model-based, SEER R-indexes reproduce standard estimates better than the empirical indexes for cervical and testicular cancers (PRD approaching zero with growing registration time).

A complete picture of percent relative differences between alternative and standard complete prevalence estimates is given in **Tables 3A, B** (EU Emp vs EU mod) and **Tables 4A, B** (SEER mod Vs EU mod), as a function of the duration of registration, starting from the group of 10 registries in operation for 10-14 years to the group of 17 registries active for 25-35 years. Mean standard complete prevalence proportion and PRD values in each pool of registries are reported by sex and cancer site. Negative values of PRD indicate an average underestimation of complete prevalence compared to the standard method.

**Table 3a:** Comparison between empirical and standard model-based complete prevalence at 1/1/2013 by cancer site for the 62 European registries included in the study grouped by registration time length (from 10-14 years to 25-35 years), Men.

Registration time interval in years (number of registries)	10-14 y (10)		15-19 y (12)		20-24 y (13)		25-35 y (17)		Registration time length with PRD≤ 5%
Cancer site	Standard complete prev	PRD	Standard complete prev	PRD	Standard complete prev	PRD	Standard complete prev	PRD	
	Prop *100,000	*%*	Prop *100,000	*%*	Prop *100,000	*%*	Prop *100,000	*%*	*number of years*
**All cancers**	3,476	*-7.1*	3,757	*-4.5*	3,747	*-3.3*	3,692	*-2.2*	>15
**Prostate**	959	*-2.8*	1,190	*-1.5*	1,450	*-0.5*	1,225	*0.0*	>10
**Colon Rectum**	539	*-3.0*	565	*-2.0*	501	*-1.3*	550	*-0.8*	>10
**Bladder**	453	*-2.2*	456	*-2.1*	281	*-2.1*	462	*-1.9*	>10
**Non-Hodgkin lymphoma**	232	*-5.4*	251	*-4.8*	247	*-4.5*	245	*-4.0*	>15
**Lung**	195	*-7.3*	197	*-4.6*	180	*-2.3*	171	*-1.1*	>15
**Kidney**	182	*-5.6*	203	*-3.1*	173	*-2.2*	184	*-1.4*	>15
**Testis**	154	*-9.4*	160	*-10.5*	166	*-10.2*	167	*-8.4*	none
**Skin melanoma**	146	*-4.3*	171	*-2.7*	186	*-2.4*	213	*-2.4*	>10
**Larynx**	103	*-2.7*	97	*-3.6*	79	*-2.2*	95	*-1.5*	>10
**Stomach**	100	*-5.2*	106	*-4.2*	67	*-3.5*	96	*-2.8*	>10
**Head and Neck**	93	*-3.6*	85	*-2.4*	108	*-1.3*	89	*-0.6*	>10
**Thyroid**	89	*0.7*	79	*-0.9*	53	*-0.8*	73	*-3.9*	>10 *
**Hodgkin lymphoma**	70	*-10.6*	66	*-9.1*	59	*-8.1*	59	*-6.7*	none
**CLL/SLL**	55	*-4.2*	58	*-2.0*	64	*-1.4*	55	*-0.9*	>10
**Liver**	47	*-3.1*	49	*-1.1*	28	*-1.0*	36	*-0.7*	>10
**Brain**	45	*-14.4*	47	*-10.6*	42	*-9.7*	54	*-8.5*	none
**Multiple myeloma**	37	*-3.0*	39	*-1.3*	36	*-0.4*	38	*-0.2*	>10
**Soft tissues**	35	*-15.7*	37	*-13.8*	36	*-13.2*	37	*-10.7*	none
**Pancreas**	22	*-3.5*	24	*-0.4*	23	*0.2*	21	*-0.1*	>10
**Oesophagus**	18	*-2.0*	20	*-0.8*	28	*-0.4*	18	*0.0*	>10
**Penis**	19	*-4.5*	18	*-4.5*	17	*-3.6*	19	*-3.3*	>10
**AML**	16	*-1.6*	21	*-3.0*	16	*-3.2*	15	*-1.9*	>10
**Bones**	15	*-20.6*	18	*-20.7*	16	*-19.2*	18	*-18.3*	none
**Gallbladder**	13	*-4.1*	11	*-2.3*	10	*-0.7*	12	*-0.3*	>10
**CML**	11	*1.6*	13	*-0.7*	14	*-0.7*	12	*-0.2*	>10

CLL/SLL, Chronic lymphocytic leukaemia/small lymphocytic lymphoma; AML, Acute myeloid leukaemia; CML, Chronic myeloid leukaemia.

* for thyroid cancer values of PRD grow as registration length increases and for female thyroid cancer PRD slightly exceeds 5% after 25 years of registration (consistently with Empirical vs EU model-based R-index patterns).

Weighted average percent relative difference (PRD, %) between **empirical** and **standard model-based** complete prevalence estimates (the average is weighted using registries population). Registration time length with PRD values not exceeding 5%.

**Table 3b:** Comparison between empirical and standard model-based complete prevalence at 1/1/2013 by cancer site for the 62 European registries included in the study grouped by registration time length (from 10-14 years to 25-35 years), Women.

Registration time interval in years (number of registries)	10-14 y (10)		15-19 y (12)		20-24 y (13)		25-35 y (17)		Registration time length with PRD ≤ 5%
Cancer Site	Standard complete prev	PRD	Standard complete prev	PRD	Standard complete prev	PRD	Standard complete prev	PRD	
	Prop *100,000	*%*	Prop *100,000	*%*	Prop *100,000	*%*	Prop *100,000	*%*	*number of years*
**All cancers**	4,380	*-8.4*	4,388	*-6.7*	4,305	*-5.4*	4,369	*-4.1*	>20
**Breast**	1,700	*-4.8*	1,831	*-4.1*	1,869	*-3.0*	1,777	*-2.0*	>10
**Colon Rectum**	477	*-4.0*	485	*-3.0*	443	*-2.4*	497	*-1.7*	>10
**Thyroid**	371	*-0.4*	278	*-2.2*	217	*-3.1*	257	*-6.1*	>10 *
**Corpus Uteri**	341	*-5.4*	305	*-4.0*	328	*-2.8*	343	*-2.6*	>10
**Cervix Uteri**	268	*-21.1*	225	*-20.2*	258	*-20.5*	230	*-21.3*	none
**Non-Hodgkin lymphoma**	203	*-6.2*	209	*-5.6*	207	*-5.4*	212	*-4.5*	>20
**Skin melanoma**	185	*-8.2*	232	*-5.8*	262	*-5.4*	289	*-5.4*	>20
**Ovary**	157	*-9.2*	145	*-9.8*	153	*-8.9*	151	*-8.4*	none
**Bladder**	114	*-4.4*	120	*-4.2*	77	*-3.6*	124	*-3.2*	>10
**Kidney**	115	*-7.1*	111	*-4.6*	109	*-4.0*	110	*-2.8*	>15
**Lung**	85	*-4.7*	90	*-2.8*	81	*-1.1*	100	*-0.5*	>10
**Stomach**	79	*-4.6*	77	*-5.0*	46	*-5.2*	75	*-5.1*	>10
**Hodgkin lymphoma**	61	*-12.3*	57	*-13.2*	49	*-11.0*	52	*-9.1*	none
**CLL/SLL**	41	*-2.4*	40	*-0.9*	48	*-0.4*	42	*-0.2*	>10
**Brain**	37	*-14.4*	38	*-9.8*	32	*-9.5*	47	*-8.0*	none
**Head and Neck**	39	*-5.8*	39	*-4.4*	42	*-2.9*	44	*-2.0*	>15
**Multiple myeloma**	32	*-2.2*	33	*-1.1*	31	*-0.5*	34	*-0.1*	>10
**Soft tissues**	31	*-18.4*	31	*-16.6*	31	*-16.5*	33	*-14.6*	none
**Liver**	20	*-5.5*	17	*-2.5*	9	*-2.2*	15	*-2.3*	>15
**Pancreas**	20	*-3.4*	22	*-0.3*	20	*0.2*	20	*-0.1*	>10
**Gallbladder**	17	*-1.5*	14	*-0.6*	11	*-0.4*	13	*-0.7*	>10
**AML**	15	*-6.7*	19	*-6.1*	18	*-4.9*	15	*-4.1*	>20
**Bones**	14	*-15.0*	15	*-18.3*	15	*-18.2*	14	*-17.3*	none
**Larynx**	11	*-2.6*	12	*-4.5*	11	*-2.9*	11	*-2.5*	>10
**CML**	9	*-1.8*	10	*-1.2*	10	*-0.2*	9	*-0.5*	>10
**Oesophagus**	6	*-1.9*	7	*-0.8*	9	*-0.3*	7	*-0.5*	>10

CLL/SLL, Chronic lymphocytic leukaemia/small lymphocytic lymphoma; AML, Acute myeloid leukaemia; CML, Chronic myeloid leukaemia.

* for thyroid cancer values of PRD grow as registration length increases and for female thyroid cancer PRD slightly exceeds 5% after 25 years of registration (consistently with Empirical vs EU model-based R-index patterns).

Weighted average percent relative difference (PRD, %) between **empirical** and **standard model-based** complete prevalence estimates (the average is weighted using registries population). Registration time length with PRD values not exceeding 5%.

**Table 4a:** Comparison between SEER and standard model-based complete prevalence at 1/1/2013 by cancer site for the 62 European registries included in the study grouped by registration time length (from 10-14 years to 25-35 years), Men.

Registration time interval in years (number of registries)	10-14 y (10)		15-19 y (12)		20-24 y (13)		25-35 y (17)		Registration time length with PRD ≤ 5%
Cancer site	Standard complete prev	PRD	Standard complete prev	PRD	Standard complete prev	PRD	Standard complete prev	PRD	
	Prop *100,000	*%*	Prop *100,000	*%*	Prop *100,000	*%*	Prop *100,000	*%*	*number of years*
**All cancers**	3,476	*-1.2*	3,757	*-1.1*	3,747	*-1.3*	3,692	*-0.7*	>10
**Prostate**	959	*2.3*	1,190	*1.3*	1,450	*0.9*	1,225	*0.2*	>10
**Colon Rectum**	539	*8.7*	565	*5.3*	501	*3.8*	550	*1.3*	>15
**Bladder**	453	*7.1*	456	*4.1*	281	*2.6*	462	*0.6*	>15
**Non-Hodgkin lymphoma**	232	*-9.8*	251	*-5.0*	247	*-4.5*	245	*-2.4*	>15
**Lung**	195	*8.0*	197	*5.0*	180	*4.8*	171	*2.3*	>15
**Kidney**	182	*6.3*	203	*4.7*	173	*3.6*	184	*2.0*	>15
**Testis**	154	*7.8*	160	*5.7*	166	*3.8*	167	*1.4*	>20
**Skin melanoma**	146	*6.9*	171	*4.4*	186	*2.6*	213	*1.1*	>15
**Larynx**	103	*8.9*	97	*7.2*	79	*4.1*	95	*1.3*	>20
**Stomach**	100	*-6.8*	106	*-4.7*	67	*-2.5*	96	*-0.8*	>15
**Thyroid**	89	*20.2*	79	*14.4*	53	*10.6*	73	*5.3*	>20
**Hodgkin lymphoma**	70	*2.3*	66	*3.8*	59	*4.3*	59	*3.3*	>10
**CLL/SLL**	55	*-1.3*	58	*-0.5*	64	*-1.1*	55	*-0.8*	>10
**Liver**	47	*-0.4*	49	*-0.3*	28	*-0.6*	36	*-0.3*	>10
**Brain**	45	*3.2*	47	*4.4*	42	*4.6*	54	*3.1*	none *
**Multiple myeloma**	37	*-4.2*	39	*-1.9*	36	*-0.8*	38	*-0.1*	>10
**Soft tissue**	35	*2.6*	37	*2.4*	36	*0.8*	37	*-0.2*	>10
**Pancreas**	22	*2.1*	24	*1.9*	23	*1.7*	21	*0.7*	>10
**Oesophagus**	18	*4.9*	20	*2.1*	28	*1.6*	18	*0.4*	>10
**AML**	16	*-0.1*	21	*4.1*	16	*4.9*	15	*4.6*	>10
**Bones**	15	*1.0*	18	*-0.7*	16	*-1.6*	18	*-3.3*	>10
**Gallbladder**	13	*4.6*	11	*2.7*	10	*2.0*	12	*0.8*	>10
**CML**	11	*-8.5*	13	*-0.3*	14	*1.9*	12	*1.9*	>15

CLL/SLL, Chronic lymphocytic leukaemia/small lymphocytic lymphoma; AML, Acute myeloid leukaemia; CML, Chronic myeloid leukaemia.

* PRD for male Brain cancer reflect compensations of under/over-estimates between SEER and Standard R-index.

Weighted average percent relative difference (PRD, %) between **SEER** and **standard model-based** complete prevalence estimates (the average is weighted using registries population). Registration time length with PRD values not exceeding 5%.

**Table 4b:** Comparison between SEER and standard model-based complete prevalence at 1/1/2013 by cancer site for the 62 European registries included in the study grouped by registration time length (from 10-14 years to 25-35 years), Women.

Registration time interval in years (number of registries)	10-14 y (10)		15-19 y (12)		20-24 y (13)		25-35 y (17)		Registration time length with PRD≤ 5%
Cancer site	Standard complete prev	PRD	Standard complete prev	PRD	Standard complete prev	PRD	Standard complete prev	PRD	
	Prop *100,000	*%*	Prop *100,000	*%*	Prop *100,000	*%*	Prop *100,000	*%*	*number of years*
**All cancers**	4,380	*2.8*	4,388	*2.8*	4,305	*2.5*	4,369	*1.6*	>10
**Breast**	1,700	*0.1*	1,831	*0.5*	1,869	*0.6*	1,777	*0.3*	>10
**Colon Rectum**	477	*6.2*	485	*4.3*	443	*3.5*	497	*1.6*	>15
**Thyroid**	371	*7.1*	278	*6.2*	217	*5.3*	257	*3.0*	>20
**Corpus Uteri**	341	*22.3*	305	*14.9*	328	*9.6*	343	*3.8*	>25
**Cervix Uteri**	268	*-5.8*	225	*-2.1*	258	*-1.0*	230	*0.6*	>15
**Non-Hodgkin lymphoma**	203	*-9.1*	209	*-5.5*	207	*-4.9*	212	*-2.9*	>20
**Skin melanoma**	185	*6.6*	232	*4.9*	262	*3.7*	289	*1.9*	>15
**Ovary**	157	*4.1*	145	*3.9*	153	*3.7*	151	*2.6*	>10
**Kidney**	115	*0.9*	111	*1.4*	109	*1.4*	110	*0.7*	>10
**Bladder**	114	*5.2*	120	*3.2*	77	*2.2*	124	*0.5*	>10
**Lung**	85	*6.3*	90	*4.3*	81	*3.2*	100	*1.3*	>15
**Stomach**	79	*-13.7*	77	*-10.2*	46	*-7.4*	75	*-3.6*	>25
**Hodgkin lymphoma**	61	*2.0*	57	*2.9*	49	*3.0*	52	*2.2*	>10
**CLL/SLL**	41	0.0	40	0.9	48	0.6	42	0.3	>10
**Brain**	37	*13.3*	38	*14.8*	32	*13.1*	47	*7.7*	none
**Multiple myeloma**	32	*-1.6*	33	*0.8*	31	*1.3*	34	*0.7*	>10
**Soft tissues**	31	*-1.6*	31	*-2.2*	31	*-2.4*	33	*-1.9*	>10
**Pancreas**	20	*2.6*	22	*2.8*	20	*2.3*	20	*1.0*	>10
**Liver**	20	*7.1*	17	*4.5*	9	*3.7*	15	*1.2*	>15
**Gallbladder**	17	*-1.5*	14	*0.3*	11	*0.7*	13	*0.5*	>10
**AML**	15	*-2.5*	19	*1.9*	18	*3.3*	15	*2.8*	>10
**Bones**	14	*7.2*	15	*2.8*	15	*0.9*	14	*-3.0*	>15
**Larynx**	11	*1.4*	12	*2.0*	11	*0.8*	11	*0.1*	>10
**CML**	9	*-13.7*	10	*-3.9*	10	*-0.7*	9	*1.3*	>15
**Oesophagus**	6	*1.3*	7	*1.3*	9	*1.6*	7	*0.7*	>10

CLL/SLL, Chronic lymphocytic leukaemia/small lymphocytic lymphoma; AML, Acute myeloid leukaemia; CML, Chronic myeloid leukaemia.

Weighted average percent relative difference (PRD, %) between **SEER** and **standard model-based** complete prevalence estimates (the average is weighted using registries population). Registration time length with PRD values not exceeding 5%.

The empirical R-index underestimates compared to the gold standard (**Tables 3A, B**) but the difference declines as registration time increases. The two methods lead to similar complete prevalence (PRD not exceeding 5% in absolute value) already after 10 or 15 years of registration for most cancers of the elderly, including those at highest prevalence (breast, prostate, colon and rectum, bladder) and those at poorest prognosis (e.g. oesophagus, larynx, gallbladder, pancreas, multiple myeloma) that show the lowest discrepancies. Most tumours at early onset represent an exception to this general pattern. PRD values reach 10-20% (testis, brain, bones, soft tissues and cervical cancers, Hodgkin lymphoma) and are scarcely sensitive to the duration of registration. On the contrary, more comparable estimates were observed for skin melanoma and thyroid cancers, both at early onset and with remarkably rising incidence across Europe.

SEER R-indexes may provide either under- or over-estimations of standard complete prevalence (**Tables 4A, B**) that diminish as registration time grows. They provide similar estimates to the standard method after 10 or 15 years of registration for most tumours and, being based on models as well, even for most of early onset tumours (Hodgkin lymphoma, soft tissues, bones, cervix uteri, skin melanoma). Wider discrepancies were instead found when incidence and survival patterns in US and European populations determine differences between standard and SEER R-index values (non-Hodgkin lymphomas, thyroid, corpus uteri, testis, brain, larynx and stomach cancers). Notably PRD values (within 5%) for male brain cancer do not properly reflect the actual differences between SEER and standard R-index by age (under- and over- estimations are compensated in the weighted average) regardless of the duration of registration.

This comparative assessment of the alternative methods to derive complete cancer prevalence is summarised in **Table 5** to facilitate readability and use of the results.

**Table 5:** Summary table reporting the registration time length (years) associated to comparable complete prevalence estimates (within a tolerance lower than 5%) between alternative (Empirical or SEER model-based) and standard completeness index method.

Registration time length (years)	Sex	European Empirical	SEER Model Based
**>10**	**M**	Bladder; Colon Rectum; Gallbladder; Head and Neck; Larynx; Liver; Multiple myeloma; Oesophagus; Pancreas; Penis; Prostate; Skin melanoma; Stomach; Thyroid; CLL/SLL; AML; CML	All cancers; Bones; Gallbladder; Hodgkin lymphoma; Liver; Multiple myeloma; Oesophagus; Pancreas; Prostate; Soft tissue; CLL/SLL; AML
	**F**	Bladder; Breast; Colon Rectum; Corpus Uteri; Gallbladder; Larynx; Lung; Multiple myeloma; Oesophagus; Pancreas; Stomach; Thyroid; CLL/SLL; CML	All cancers; Bladder; Breast; Gallbladder; Hodgkin’s lymphoma; Kidney; Larynx; Multiple myeloma; Oesophagus; Ovary; Pancreas; Soft tissues; CLL/SLL; AML
**>15**	**M**	All cancers; Kidney; Lung; Non-Hodgkin lymphoma;	Bladder; Colon Rectum; Kidney; Lung; Non- Hodgkin lymphoma; Skin melanoma; Stomach; CML
	**F**	Head and Neck; Kidney	Bones; Cervix Uteri; Colon Rectum; Liver; Lung; Skin melanoma; CML
**>20**	**M**		Larynx; Testis; Thyroid
	**F**	All cancers; Non-Hodgkin lymphoma; Skin melanoma; AML	Non-Hodgkin lymphoma; Thyroid
**>25**	**M**		
	**F**		Corpus Uteri; Stomach
**None**	**M**	Bones; Brain; Hodgkin lymphoma; Soft tissues; Testis	Brain
	**F**	Bones; Brain; Cervix Uteri; Hodgkin lymphoma; Ovary; Soft tissues	Brain

CLL/SLL, Chronic lymphocytic leukaemia/small lymphocytic lymphoma; AML, Acute myeloid leukaemia; CML, Chronic myeloid leukaemia.

Results obtained from the 62 European registries included in the study.

## 4 Discussion

To our knowledge, this is the first study exploring the validity of alternative approaches to derive prevalence completeness indexes. The study relies on an exceptionally wide European population-based dataset covering 50% of the population of the 27 countries involved.

Model-based R-indexes were introduced more than 20 years ago (1). Nowadays observations of cancer prevalence are available for time series and populations of much greater extension, thus testing the validity of empirical indexes that have now become available is relevant for a wider application of the method. The completeness index method is indeed particularly suited for local registry-based applications that rely on the available observed limited-duration prevalence.

Other methods to estimate complete prevalence include those modelling prevalence as a function of cancer-specific incidence and survival, both derived from cancer registries’ data. Unlike the completeness index method, these methods do not rely on observed limited-duration prevalence and are more suited to derive time projections of cancer prevalence or national estimates in countries with partial registration coverage (15–18, 25).

From the validation study, a registration time period of at least 10 years turned out to be necessary to safely apply the prevalence completeness index method, confirming this cut-off as a general recommendation.

In many situations empirical R-index was found to provide complete prevalence estimates comparable to the “gold-standard”. Registries’ observation time window, cancer specific incidence age profile and prognosis act as modulating factors. For tumours mainly diagnosed in the elderly, EU empirical and EU model-based R-indexes led to similar results (within an average tolerance of 5%) when applied to prevalence data observed for at least 10 years.

By contrast, the empirical method underestimates very long-term survivorship for tumours with early age at onset, even when based on 35 years of observations. For this specific class of neoplasms, model-based methods are structurally more suited to capture unobserved survivors in the very long term. This limitation is also reflected in the estimation of all cancers that include a non-negligible proportion of juvenile cancers.

Using model-based completeness indexes derived from external rather than local patients’ populations (SEER versus European) led to comparable prevalence estimates for the majority of cancers, even when applied to minimum registration periods (10 years). The list includes also most of the early onset tumours and, as a consequence, the complex of all cancer sites. Notable discrepancies were instead observed as a result of geographical differences in cancer incidence and survival patterns, regardless of the natural history of the disease (age at onset and prognosis). This, for instance, is the case of endometrial and thyroid cancers, or of brain tumours, as the inclusion criteria of non-malignant entities may vary between SEER and European registries, thus affecting the consistency of estimates.

The results we obtained were coherent with the patterns of the relevant factors influencing cancer prevalence, e.g. age at prevalence date, low to high cancer prognosis, incidence age profile, length of the registration time period.

European model-based R-index values were slightly higher than those estimated from SEER data consistently with the prognostic differences between European and USA cancer patients, the latter generally reported to present more favourable survival levels (31). Differences are also partly due to incidence modelling choices. SEER R-indexes were indeed often derived by adopting exponential rather than polynomial incidence models (4). Finally, differences between IARC and SEER rules for identifying multiple primary tumours could also have an impact.

Parametric mixed cure models of Weibull type were used for modelling survival (1, 2). More flexible cure fraction models could have been considered (32, 33) but the choice is limited to Weibull or exponential types in the COMPREV software.

The empirical indexes were derived by pooling data of 8 European registries with available 35-year observed prevalence at the index date. The limit at 35-years is arbitrary and just reflects the maximum available time span in the EUROCARE-6 dataset. However, it has been proven to provide a sufficient basis to estimate complete cancer prevalence for major cancers and for a variety of less frequent tumours with late age at onset. Lower values might be critical and extending this limit in applications to more recent prevalence index dates is advisable, considering the continuous progresses of cancer survival over time and the availability of longer registration time series.

Empirical indexes were subject to random fluctuations when based on sparse cases, for instance in correspondence of young age at prevalence date for tumours at late onset like pancreatic or prostatic cancer. However, such fluctuations are of scarce practical relevance because the index is applied to values of observed prevalence which are almost null in these circumstances.

R-indexes were generally positively validated on a fully independent dataset of 20 registries, therefore showing that the estimation datasets used to derive model-based completeness indexes were sufficiently representative of the prevalence patterns in other European populations. However, we cannot exclude that for some neoplasms the geographical heterogeneity of incidence or prognosis may have required area-specific R-indexes.

Notably the empirical completeness R-indexes are easy to compute but inevitably refer to a specific point in time (the index date of the maximum observable cancer prevalence). Thus they must be computed on a date which is reasonably close to the index date of the limited-duration prevalence we want to complete.

Conversely model-based R-indexes require higher computational effort to model incidence and relative survival trends, but they dynamically evolve over time (the period of diagnosis is parameterised in the models) and R-index values for varying prevalence index dates can be derived through the Comprev software (4).

In conclusion, the study tests the feasibility of using alternative formulations of the completeness index method to integrate limited-duration prevalence measured by population-based cancer registries. We focused on the European context where the lack of systematic data on the overall number of cancer survivors in many countries hinders the planning of health services and particularly survivorship care planning. This appears even more limiting in light of the future scenario in which the population of cancer survivors is indeed expected to increase significantly due to ongoing demographic changes and continued advances in therapies and diagnosis. Our results may facilitate the use and dissemination of complete cancer prevalence estimates across Europe and help to close the present information gaps.

## Data availability statement

The datasets of empirical and standard model-based completeness indexes presented in this article are not readily available. Requests to access the datasets should be directed to RA, roberta.deangelis@iss.it.

## Ethics statement

Ethical review and approval was not required for the study on human participants in accordance with the local legislation and institutional requirements. Written informed consent from the participants’ legal guardian/next of kin was not required to participate in this study in accordance with the national legislation and the institutional requirements.

## Author contributions

ED carried out the study and analysed the incidence and prevalence data. SR did quality checks, prepared the SEER*Stat study database and analysed the survival data. LV analysed the survival data. CB implemented the procedures to check the raw data and to generate the SEER*Stat study database. LM, SG, AK, SL, and VJ provided advice and revised the results. RA drafted the article, designed the study and the data quality checks. The EUROCARE-6 Working Group collected, prepared, and transmitted raw data for the study database, corrected data after quality controls, checked the results of the analyses and revised the final draft of the article. All authors interpreted results. All authors contributed to the article and approved the submitted version.

## EUROCARE-6 working group

Austria: M. Hackl *(National CR)*; Belgium: E. Van Eycken; N. Van Damme *(National CR)*; Bulgaria: Z. Valerianova *(National CR)*; Croatia: M. Sekerija *(National CR)*; Cyprus: V. Scoutellas; A. Demetriou *(National CR)*; Czech Republic: L. Dušek; D. Krejici *(National CR)*; Denmark: H. Storm *(National CR)*; Estonia: M. Mägi; K. Innos* *(National CR)*; Finland: N. Malila; J. Pitkäniemi *(National CR)*; France: M. Velten *(Bas Rhin CR)*; X. Troussard *(Basse Normandie, Haematological Malignancies CR)*; A.M. Bouvier; V. Jooste* *(Burgundy, Digestive CR)*; A.V. Guizard *(Calvados, General CR)*; G. Launoy *(Calvados, Digestive CR)*; S. Dabakuyo Yonli *(Cote dOr, Gynaecologic (Breast) CR)*; M. Maynadié *(Cote dOr, Haematological Malignancies CR)*; A.S. Woronoff *(Doubs CR)*; J.B. Nousbaum *(Finistere, Digestive CR)*; G. Coureau *(Gironde, General CR)*; A. Monnereau* *(Gironde, Haematological Malignancies CR)*; I. Baldi *(Gironde, Central Nervous System CR)*; K. Hammas *(Haut-Rhin CR)*; B. Tretarre *(Herault CR)*; M. Colonna *(Isere CR)*; S. Plouvier *(Lille Area CR)*; T. D’Almeida *(Limousin CR)*; F. Molinié; A. Cowppli-Bony *(Loire-Atlantique/Vendée CR)*; S. Bara *(Manche CR)*; A. Debreuve *(Marne-Ardennes, Thyroid CR)*; G. Defossez *(Poitou-Charentes CR)*; B. Lapôtre-Ledoux *(Somme CR)*; P. Grosclaude; L. Daubisse-Marliac *(Tarn CR)*; Germany: S. Luttmann *(Bremen CR)*; R. Stabenow *(Common CR of 4 Federal States (Brandenburg, Mecklenburg-West Pomerania, Saxony-Anhalt, Thüringen))*; A. Nennecke *(Hamburg CR)*; J. Kieschke *(Lower Saxony CR)*; S. Zeissig *(Rhineland-Palatinate CR)*; B. Holleczek *(Saarland CR)*; A. Katalinic* *(Schleswig-Holstein CR)*; Iceland: H. Birgisson *(National CR)*; Ireland: D. Murray; P.M. Walsh *(National CR)*; Italy: G. Mazzoleni; F. Vittadello *(Alto Adige CR)*; F. Cuccaro *(Barletta-Andria-Trani CR)*; R. Galasso *(Basilicata CR)*; G. Sampietro *(Bergamo CR)*; S. Rosso *(Biella CR)*; C. Gasparotti; G. Maifredi *(Brescia CR)*; M. Ferrante; R. Ragusa *(Catania-Messina-Enna CR)*; A. Sutera Sardo *(Catanzaro CR)*; M.L. Gambino; M. Lanzoni *(Province of Varese and Como CR)*; P. Ballotari; E. Giacomazzi *(Cremona and Mantova CR)*; S. Ferretti *(Ferrara CR)*; A. Caldarella; G. Manneschi *(Firenze-Prato CR)*; G. Gatta*; M. Sant*; P. Baili*; F. Berrino*; L. Botta; A. Trama; R. Lillini; A. Bernasconi; S. Bonfarnuzzo; C. Vener; F. Didonè; P. Lasalvia; G. Del Monego; L. Buratti; G. Tagliabue *(Fondazione IRCCS Istituto Nazionale dei Tumori, Milan)*; D. Serraino; L. Dal Maso *(Centro di Riferimento Oncologico, IRCCS, Aviano for the Friuli Venezia Giulia CR)*; R. Capocaccia* *(Epidemiologia & Prevenzione Board)*; R. De Angelis*; E. Demuru; C. Di Benedetto; S. Rossi*; M. Santaquilani; S. Venanzi; M. Tallon *(Istituto Superiore di Sanità, Rome)*; L. Boni *(Genova CR)*; S. Iacovacci *(Latina CR)*; V. Gennaro *(Liguria, mesotheliomas CR)*; A.G. Russo; F. Gervasi *(Province of Milan and Lodi CR)*; G. Spagnoli *(Modena CR)*; L. Cavalieri d’Oro *(Monza and Brianza CR)*; M. Fusco; M.F. Vitale *(Napoli 3 South CR)*; M. Usala *(Nuoro CR)*; W. Mazzucco *(Palermo CR)*; M. Michiara *(Parma CR)*; G. Chiranda *(Piacenza CR)*; G. Cascone; C.P. Rollo *(Ragusa CR)*; L. Mangone *(Reggio Emilia CR)*; F. Falcini *(Romagna CR)*; R. Cavallo *(Salerno CR)*; D. Piras *(Sassari CR)*; A. Madeddu; F. Bella *(Siracusa CR)*; A.C. Fanetti *(Sondrio CR)*; S. Minerba *(Taranto CR)*; G. Candela; T. Scuderi *(Trapani CR)*; R.V. Rizzello *(Trento CR)*; F. Stracci *(Umbria CR)*; M. Rugge *(Veneto CR)*; A. Brustolin *(Viterbo CR)*; Latvia: S. Pildava *(National CR)*; Lithuania: G. Smailyte *(National CR)*; Malta: M. Azzopardi *(National CR)*; Norway: T.B. Johannesen* *(National CR)*; Poland: J. Didkowska; U. Wojciechowska *(National CR)*; M. Bielska-Lasota* *(National Institute of Public Health-National Institute of Hygiene-National Research Institute, Warsaw)*; Portugal: A. Pais *(Central Portugal CR)*; J. Rodrigues; M.J. Bento *(Northern Portugal CR)*; A. Miranda *(Southern Portugal CR)*; Slovakia: C. Safaei Diba *(National CR)*; Slovenia: V. Zadnik; T. Zagar (National CR); Spain: C. Sánchez-Contador Escudero; P. Franch Sureda *(Balearic Islands, Mallorca CR)*; A. Lopez de Munain; M. De-La-Cruz *(Basque Country CR)*; M.D. Rojas; A. Aleman *(Canary Islands CR)*; A. Vizcaino *(Castellon CR)*; R. Marcos-Gragera; A. Sanvisens *(Girona CR)*; M.J. Sanchez *(Granada CR)*; M.D. Chirlaque; A. Sanchez-Gil *(Murcia CR)*; M. Guevara*; E. Ardanaz *(Navarra CR, CIBERESP)*; A. Ameijide; M. Carulla *(Tarragona CR)*; Switzerland: Y. Bergeron *(Fribourg CR)*; C. Bouchardy *(Geneva CR)*; S. Mohsen Mousavi; P. Went *(Graubünden and Glarus CR)*; S. Mohsen Mousavi; M. Blum *(Eastern Switzerland CR)*; A. Bordoni *(Ticino CR)*; The Netherlands: O. Visser* *(National CR)*; UK-England: S. Stevens; J. Broggio *(National CR)*; UK-Northern Ireland: A. Gavin* *(National CR)*; UK-Scotland: D. Morrison *(National CR)*; UK-Wales: D. W. Huws* *(National CR).* *EUROCARE Steering Committee.
