# Supplementary material for: Estimating complete cancer prevalence in Europe: validity of alternative vs standard completeness indexes
Source: Front Oncol. 2023 Apr 24;13:1114701. doi: 10.3389/fonc.2023.1114701 (PMC10166634; doi:10.3389/fonc.2023.1114701)

**Supplementary Materials**

**Table A. 1 -** Definition of malignant cancer entities included in the study and age strata included in survival models

| **Cancer entities** | **Detailed description** | **ICD-O-3 Topography** | **ICD-O-3 Morphology** | **Age strata in survival models** |
| --- | --- | --- | --- | --- |
| All cancers | All sites excluding non-melanoma skin cancer | All Sites from SEER Site recode (https://seer.cancer.gov/siterecode/) | | 0-14, 15-44, 45-54, 55-64, 65-74;75+ |
| Head and neck | Tongue, gum, floor of mouth, other and unspecified mouth, oropharynx, nasopharynx, hypopharynx, other oral cavity and pharynx | C01-C06,C09-C14 | excluding 9050-9055, 9140, 9590-9992 | 0-44, 45-54, 55-64, 65-74, 75+ |
| Oesophagus |  | C15 | excluding 9050-9055, 9140, 9590-9992 | 0-44, 45-54, 55-64, 65-74, 75+ |
| Stomach |  | C16 | excluding 9050-9055, 9140, 9590-9992 | 0-44, 45-54, 55-64, 65-74, 75+ |
| Colon and rectum | Colon, rectum, rectosigmoid junction, anal canal, anus and intestine NOS | C18-C21,C26.0 | excluding 9050-9055, 9140, 9590-9992 | 0-44, 45-54, 55-64, 65-74, 75+ |
| Liver | Liver and intrahepatic bile ducts (excluding metastatic and uncertain behaviour) | C22 | excluding 9050-9055, 9140, 9590-9992 | 0-44, 45-54, 55-64, 65-74, 75+ |
| Gallbladder | Gallbladder, ampulla of Vater and extrahepatic bile ducts | C23-C24 | excluding 9050-9055, 9140, 9590-9992 | 0-44, 45-54, 55-64, 65-74, 75+ |
| Pancreas |  | C25 | excluding 9050-9055, 9140, 9590-9992 | 0-44, 45-54, 55-64, 65-74, 75+ |
| Larynx |  | C32 | excluding 9050-9055, 9140, 9590-9992 | 0-99 |
| Lung | Trachea, bronchus and lung (excluding mesotheliomas) | C33.9,C34 | excluding 9050-9055, 9140, 9590-9992 | 0-44, 45-54, 55-64, 65-74, 75+ |
| Bone | Bones, joints and articular cartilage | C40-C41 | excluding 9050-9055, 9140, 9590-9992 | 0-99 |
| Soft tissue | Connective subcutaneous and other soft tissue (including heart) | C38.0,C47,C49 | excluding 9050-9055, 9140, 9590-9992 | 0-39; 40+ |
| Skin melanoma |  | C44 | 8720-8790 | 0-44, 45-54, 55-64, 65-74, 75+ |
| Breast | Female breast | C500-C509 | excluding 9050-9055, 9140, 9590-9992 | 0-44, 45-54, 55-64, 65-74, 75+ |
| Cervix uteri |  | C53 | excluding 9050-9055, 9140, 9590-9992 | 0-44, 45-54, 55-64, 65-74, 75+ |
| Corpus uteri | Corpus, isthmus, other | C54 | excluding 9050-9055, 9140, 9590-9992 | 0-44, 45-54, 55-64, 65-74, 75+ |
| Ovary | Ovary and other uterine adnexa | C56.9, C57.0-C57.4, C57.7 | excluding 9050-9055, 9140, 9590-9992 | 0-44, 45-54, 55-64, 65-74, 75+ |
| Prostate |  | C619 | excluding 9050-9055, 9140, 9590-9992 | 0-99 |
| Testis |  | C62 | excluding 9050-9055, 9140, 9590-9992 | 0-44, 45-54, 55-64, 65+ |
| Penis | Penis and other male genital organs | C60, C63 | excluding 9050-9055, 9140, 9590-9992 | 0-99 |
| Urinary bladder | Urinary bladder (including benign, uncertain and in situ neoplasms) | C67 | excluding 9050-9055, 9140, 9590-9992 | 0-44, 45-54, 55-64, 65-74, 75+ |
| Kidney | Kidney and other and unspecified urinary organs (excluding bladder) | C64-C66, C68 | excluding 9050-9055, 9140, 9590-9992 | 0-44, 45-54, 55-64, 65-74, 75+ |
| Brain | Excluding meningiomas (including benign and uncertain neoplasms) | C71 | excluding 9050-9055, 9140, 9530-9539, 9590-9992 | 0-14, 15-44, 45-54, 55-64, 65-74;75+ |
| Thyroid |  | C73.9 | excluding 9050-9055, 9140, 9590-9992 | 0-44, 45-54, 55-64, 65-74, 75+ |
| Multiple myeloma | Multiple myeloma, plasma cell leukaemia, plasmacytoma NOS, plasmacytoma extramedullary |  | 9731-9734 | 0-44, 45-54, 55-64, 65-74, 75+ |
| Hodgkin's lymphoma |  |  | 9650-9667 | 0-14, 15-44, 45-54, 55-64, 65-74;75+ |
| Non-Hodgkin lymphoma | Malignant lymphomas NOS or diffuse, mature B-cell lymphomas, mature T- and NK-cell lymphomas, adult T-cell leukaemia/lymphoma (HTLV-1 positive) |  | 9590-9596, 9671, 9673, 9675, 9678-9680, 9684, 9687, 9689-9691, 9695, 9698-9702, 9705, 9708-9709, 9714-9719, 9761, 9826, 9827 | 0-99 |
| Chronic lymphocytic leukaemia/small lymphocytic lymphoma (CLL/SLL) | Small B-cell lymphocytic lymphoma, B-cell chronic lymphocytic leukaemia/small lymphocytic lymphoma |  | 9670, 9823 | Chronic lymphocytic leukaemia/small lymphocytic lymphoma (CLL/SLL) |
| Acute myeloid leukaemia (AML) | Acute myeloid leukaemia |  | 9840, 9861, 9866-9867, 9870-9874, 9891, 9895-9897, 9898, 9910, 9920, 9930-9931, 9984, 9987 | Acute myeloid leukaemia (AML) |
| Chronic myeloid leukaemia (CML) | Chronic myeloid leukaemia |  | 9863, 9875 | Chronic myeloid leukaemia (CML) |

**Figure** **A. 1** - Fit of Weibull cure models: observed Vs predicted relative survival over the period of follow-up by cancer site, sex, age and period of diagnosis. Selected examples.


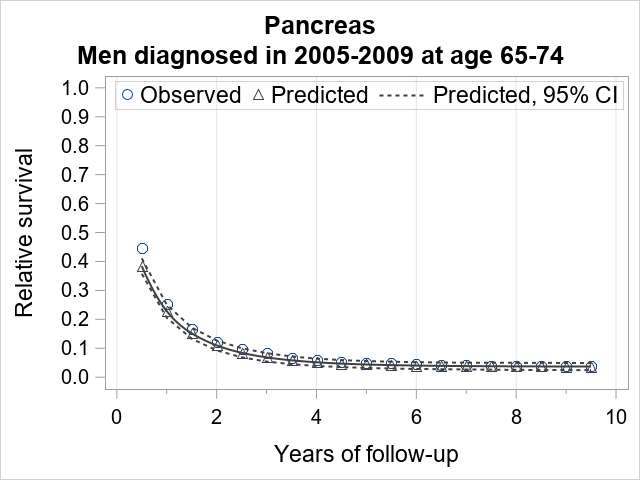

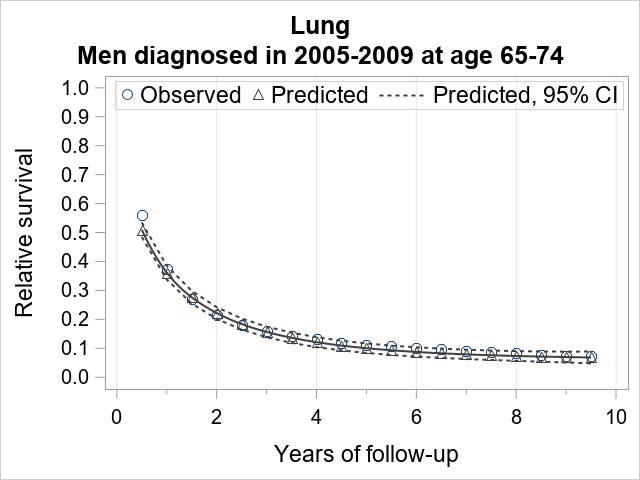


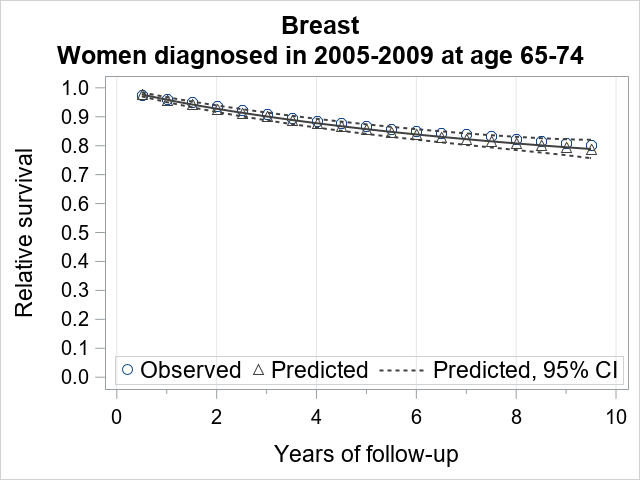

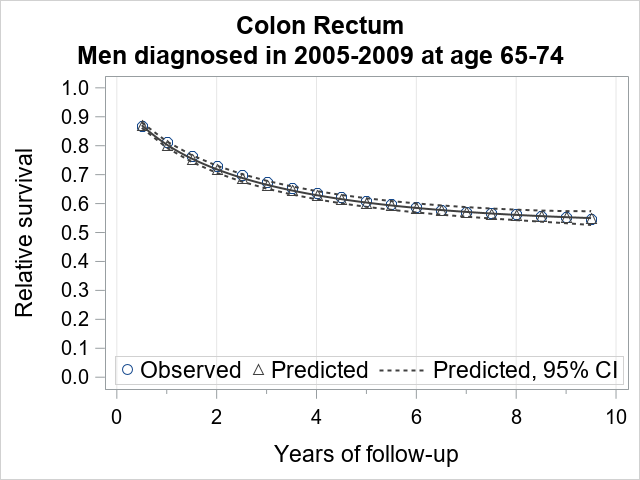


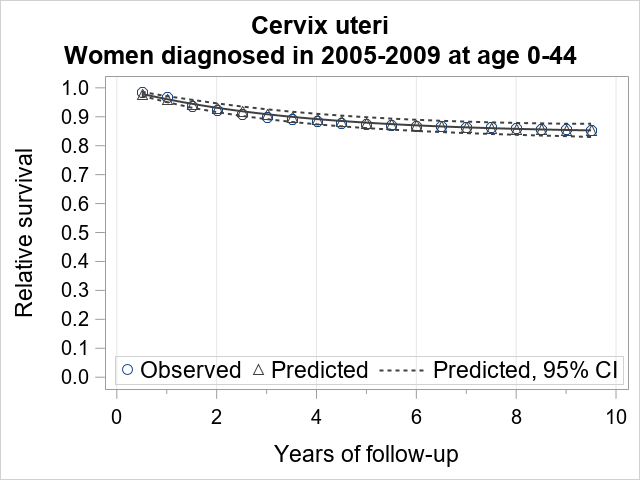

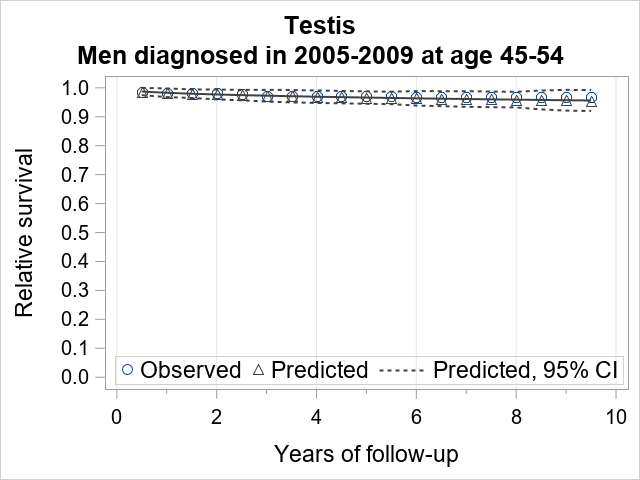


**Figure A. 2** - Fit of incidence regression models: observed Vs estimated exponential and polynomial estimates of incidence rates (values per 100,000) by cancer site, sex, age and period of diagnosis. Selected examples.


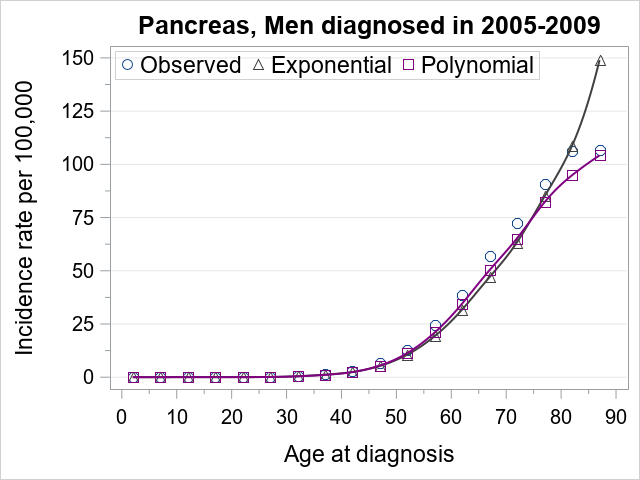

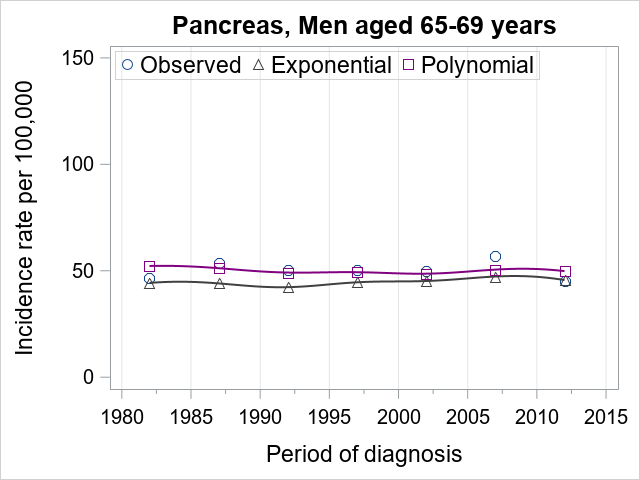


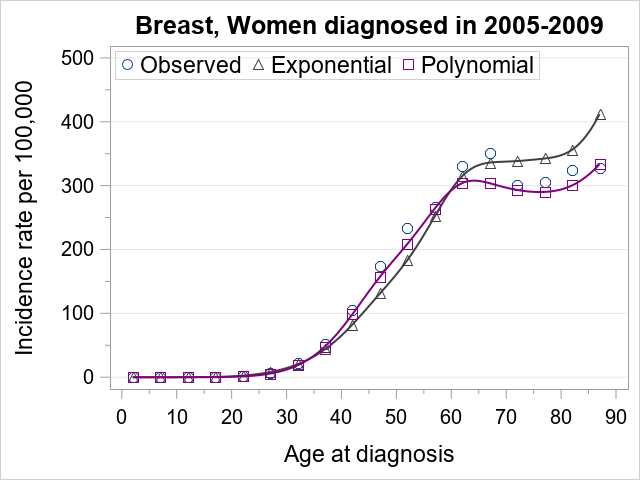

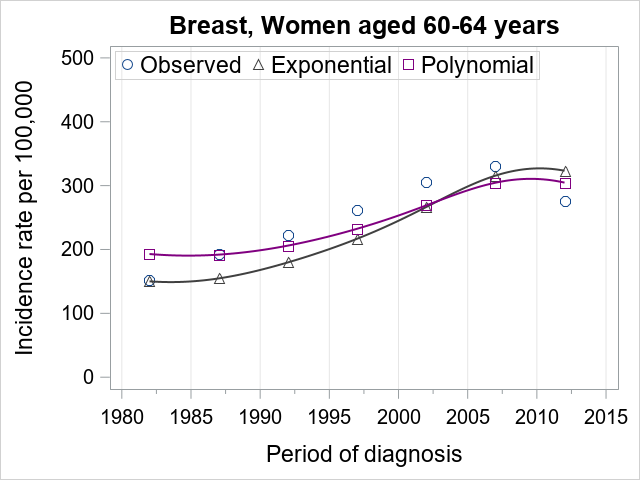


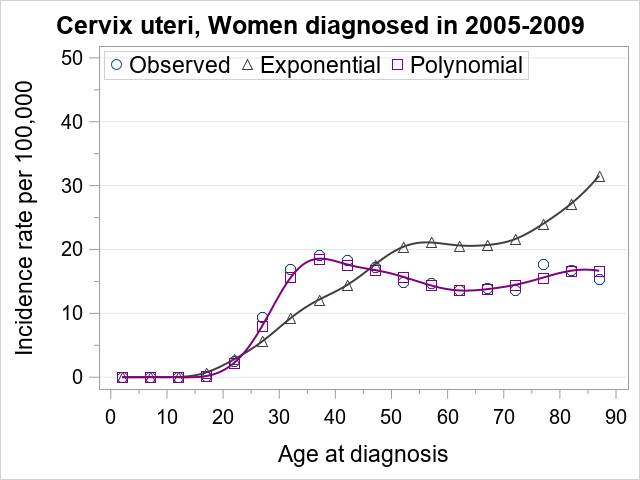

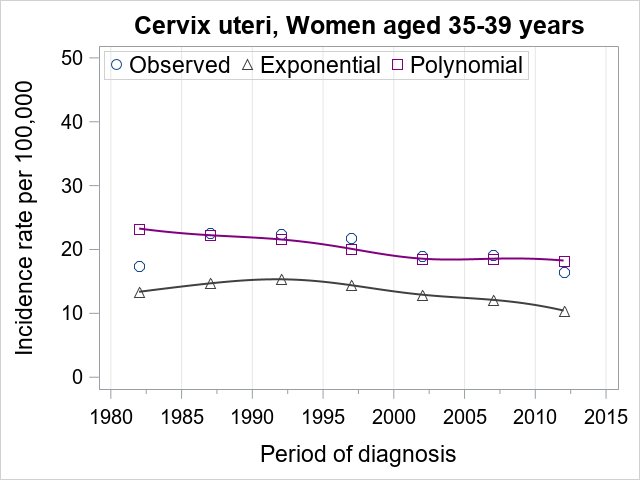

Supplement: Supplementary file 1 [file DataSheet_1.docx]
